# Withholding and withdrawing life-sustaining treatment: a comparative study of the ethical reasoning of physicians and the general public

**DOI:** 10.1186/cc6786

**Published:** 2008-02-15

**Authors:** Anders Rydvall, Niels Lynöe

**Affiliations:** 1 Department of Surgical and Perioperative Sciences, Anaesthesiology, University Hospital of Northern Sweden, Lasarettsbacken SE-90185 Umeå, Sweden; 2 Department of Learning, Informatics, Management and Ethics, Karolinska Institutet, Berzelius vaeg 3 SE-17177 Stockholm, Sweden

## Abstract

**Background:**

Our objective was to investigate whether a consensus exists between the general public and health care providers regarding the reasoning and values at stake on the subject of life-sustaining treatment.

**Methods:**

A postal questionnaire was sent to a random sample of members of the adult population (*n *= 989) and to a random sample of intensive care doctors and neurosurgeons (*n *= 410) practicing in Sweden in 2004. The questionnaire was based on a case involving a severely ill patient and presented arguments for and against withholding and withdrawing treatment, and providing treatment that might hasten death.

**Results:**

Approximately 70% of the physicians and 51% of the general public responded. A majority of doctors (82.3%) stated that they would withhold treatment, whereas a minority of the general public (40.2%) would do so; the arguments forwarded (for instance, belief that the first task of health care is to save life) and considerations regarding quality of life differed significantly between the two groups. Most physicians (94.1%) and members of the general public (77.7%) were prepared to withdraw treatment, and most (95.1% of physicians and 82% of members of the general public) agreed that sedation should be provided.

**Conclusion:**

There are indeed considerable differences in how physicians and the general public assess and reason in critical care situations, but the more hopelessly ill the patient became the more the groups' assessments tended to converge, although they prioritized different arguments. In order to avoid unnecessary dispute and miscommunication, it is important that health care providers be aware of the public's views, expectations, and preferences.

## Introduction

Health care providers have a long tradition of ethical reasoning, which is evolving continuously alongside the development of modern medicine. The overall assumption is that the primary task of health care providers is to promote health and, whenever possible, to save lives and alleviate suffering. Health care providers are also supposed to avoid harming patients when they provide treatment. Finally, they are expected to respect a patient's autonomy and integrity as well as the principle of justice, which requires all to be treated equally.

However, when a patient is unable to make decisions, who should do so in their place – doctors or relatives? Alternatively, should we adhere to the patient's previously oral or written directives, or to a hypothetical judgement of what the patient would have preferred if they had been able to describe their preferences [1-3]? It is generally accepted that relatives make surrogate decisions that are in the patient's best interests. Different relatives might have different opinions, however, as might different doctors and nurses [4-7]. Moreover, there are differences between countries in terms of, for instance, respecting advance directives [8-10]. Health care providers may hope and sometimes presume that their ethics and reasoning are endorsed by the general public, and accordingly that a consensus does indeed exist. However, several studies have indicated that this is not always the case; members of the general public and physicians appear to differ in their perspectives on the role of relatives and others in the decision making process regarding care for terminally ill patients [11].

Differences in attitude, reasoning and judgement result from divergent systems of values or interpretation of empirical data, and from use of different methodological approaches that may focus on alternative aspects [12]. The objective of the present survey was to evaluate the specific arguments, values at stake and the degree to which consensus exists in the critical care setting.

## Materials and methods

The study was conducted during the Autumn of 2004 and included a multidisciplinary group of doctors (112 neurosurgeons and 298 intensive care doctors) and a random sample of the adult population (*n *= 989) of the county of Västerbotten in northern Sweden. All participants were sent a postal questionnaire based on the case of a 72-year-old patient suffering from a severe intracerebral haemorrhage (for details, see Table 1). The case description was presented to both groups using the same, plain language. Two reminders were sent at 2-week intervals.

**Table 1:** Case description and arguments presented

Case description	Arguments^a^
**Situation A: **A previously healthy 72-year-old woman is brought to the emergency room in a deep coma for what is believed to be a stroke with a right-sided hemiphlegia. In order to conduct a CT scan and to secure respiratory function, it is necessary to intubate and mechanically ventilate the patient. The CT scan shows a large haemorrhage in the left central part of the brain. A surgical evacuation in this delicate area is considered undesirable. However, without neurosurgery, intracranial pressure will probably increase, and a herniation of the brain will occur. Accordingly, without treatment, the patient is presumed fated to die within a few days.	In favour of surgery: • Surgery should be performed because it is the first task of health care to safe lives • A neurosurgeon refers to experience from a successful case 2 years ago; thus, the surgery should be performed. • Surgery should be performed because otherwise it might be interpreted as a kind of euthanasia • Surgery should be performed because a son has asked the doctor to do everything to save his mother's life Against surgery: • Surgery should be avoided because the patient's quality of life would be greatly reduced • Surgery should be avoided because of the age of the patient • Surgery should be avoided because of the cost and uncertain result • Surgery should be avoided because of the patient's wish not to end up in a persistent vegetative state
**Situation B: **Neurosurgery has been performed and the patient is transferred to the intensive care unit. After 2 days the patient is still on the ventilator, no improvement has been observed and the patient is still deeply unconscious. After 10 days a new CT scan is conducted, which indicates that a large area of the brain is incarcerated. The patient is no longer able to breathe without a ventilator, and the physicians discuss whether to continue the treatment	In favour of continuation of ventilation: • Ventilator treatment should be continued because discontinuing it might be perceived as a kind of euthanasia • The patient's son is strongly against discontinuing ventilator treatment, thus, treatment should be continued Against continuation of ventilation: • The treatment should be discontinued because it only prolongs the dying process • The treatment should be discontinued because it is in accordance with the wishes of the patient
**Situation C: **The physicians have now decided to withdraw ventilator treatment and inform the relatives. After 12 hours of breathing unaided, the patient develops convulsions and forced breathing. The condition looks painful and stressful. In order to alleviate the patient's symptom, morphine and tranquillizers may be provided. However, these drugs might also affect the respiratory centre in the brain and accordingly hasten death	In favour of morphine and tranquillizers: • Tranquillizers and morphine should be provided in order to keep the patient free from symptoms even though they might hasten death • Tranquillizers and morphine should be provided in order to shorten the dying process Against morphine and tranquillizers: • Tranquillizers and morphine should be provided but without risking acceleration of death • Tranquillizers and morphine should not be provided if the purpose is to hasten the dying process

^a^Responders were asked to score the arguments as 'agree entirely', 'agree mostly', 'disagree mostly' or 'disagree entirely'. Afterwards, responders were asked to identify which of the arguments they deemed to be the most important (see Table II for situation A, Table III for situation B, and Table IV for situation C). CT, computed tomography

The case is developed step by step in the questionnaire text. Initially, arguments are presented for and against performing neurosurgery (Table 2). The focus then shifts to after the surgery. One week postoperatively it becomes clear that the patient will not survive; arguments for and against withdrawing life-supporting treatment are provided (Table 3). Finally, the patient is disconnected from the ventilator. In order to keep the patient calm and free from pain, they are treated with potent sedatives and analgesics, despite the risk that this will hasten death; arguments for and against this approach are provided (Table 4). The arguments presented were based on experience gained from similar cases in an intensive care unit.

**Table 2:** Responses regarding whether neurosurgery should be performed

Argument	Doctors/public	Percentage (CI)	Priority (%)
Surgery should be performed because it is the first task of health care to safe lives	Doctors Public	12.9 (9.0–16.8) 78.3 (74.7–81.9)	4.5% 29.8%
A neurosurgeon refers to experience from a successful case two years ago; thus the surgery should be performed	Doctors Public	25.0 (20.0–30.0) 80.8 (77.3–84.3)	11.1% 23.6%
Surgery should be performed because otherwise it might be interpreted as a kind of euthanasia	Doctors Public	5.6 (2.9–8.3) 55.4 (51.0–59.8)	1.2% 2.5%
Surgery should be performed because the son has asked the doctor to do anything to save his mother's life	Doctors Public	8.7 (5.4–12.0) 58.9 (54.5–63.3)	0.5% 3.9%
Surgery should be avoided since the patient's quality of life would be greatly reduced	Doctors Public	82.8 (78.5–87.1) 40.6 (36.3–44.9)	61.5% 12.5%
Surgery should be avoided due to the age of the patient	Doctors Public	18.8 (14.3–23.3) 18.2 (14.8–21.6)	1.6% 2.8%
Surgery should be avoided due to the cost and the uncertain result	Doctors Public	15.8 (11.6–20.0) 15.7 (12.5–18.9)	0.8% 2.3%
Surgery should be avoided due to the patient's wish not to end up in a persistent vegetative state	Doctors Public	71.6 (66.3–76.9) 54.5 (50.1–58.9)	18.4% 22.6%

This table shows the response pattern of the doctors and members of the general public who answered the question regrding whether neurosurgery should be performed in a formerly healthy 72-year-old patient suffering from a major haemorrhage in the left central part of the brain. The results are presented as proportions of those who agreed 'mostly' or 'entirely', with a 95% confidence interval (CI). The percentages of those who considered the argument to be the most important are also presented

**Table 3:** Responses regarding whether to continue ventilator treatment

Argument	Doctors/public	Percentage (CI)	Priority (%)
**Ventilator treatment should be continued because discontinuing it might be perceived as a form of euthanasia**	Doctors General public	6.3 (3.5–9.1) 28.3 (24.3–32.3)	5.5% 14.9%
A son is strongly against discontinuing ventilator treatment, thus treatment should be continued	Doctors General public	10.1 (6.6–13.6) 35.2 (31.0–39.4)	0.4% 7.4%
The treatment should be discontinued because it only prolongs the death process	Doctors General public	91.9 (88.9–94.9) 81.5 (78.1–84.9)	73% 42.5%
The treatment should be discontinued because it is in accordance with the wishes of the patient	Doctors General public	83.9 (79.6–88.2) 76.2 (72.4–80.0)	21.1% 35.2%

This table shows the response pattern of the doctors and members of the general public who answered the question regarding whether to continue ventilator treatment in a terminally ill patient after unsuccessful neurosurgical treatment. The results are presented as proportions of those who agreed 'mostly' or 'entirely', with a 95% confidence interval (CI). The percentages of those who considered the argument to be the most important are also presented.

**Table 4:** Responses regarding whether to administer tranquillizers and morphine

Argument	Doctors/public	Percentage (CI)	Priority (%)
**Tranquillizers and morphine should be provided in order to keep the patient free of symptom even though it might hasten death**	Doctors General public	97.6 (95.8–99.4) 95.9 (94.2–97.6)	94.4% 76.2%
Tranquillizers and morphine should be provided in order to shorten the dying process	Doctors General public	9.9 (6.4–13.4) 45.7 (41.3–50.1)	0.7% 5.8%
Tranquillizers and morphine should be provided but without risking the acceleration of death	Doctors General public	29.6 (24.3–34.9) 49.2 (44.8–53.6)	1.9% 12.2%
Tranquillizers and morphine should not be provided if the purpose is to hasten the dying process	Doctors General public	72.7 (66.5–77.9) 52.0 (47.6–56.4)	3.0% 5.8%

This table shows the response pattern of the doctors and members of the general public who answered the question regarding whether to provide tranquillizers and morphine to a terminally ill patient disconnected from life-sustaining ventilator treatment. The results are presented as proportions of those who agreed 'mostly' or 'entirely', with a 95% confidence interval (CI). The percentages of those who considered the argument to be the most important are also presented.

The respondents answered in terms of 'agreeing entirely', 'agreeing mostly', 'disagreeing mostly' and 'disagreeing entirely'. They then indicated which of the provided arguments they perceived to be the most important. The results are presented as proportions of those who agreed mostly or entirely (with 95% confidence interval) and a priority list of arguments regarding to be the most important. Similar to performing a hypothesis test, a 95% confidence interval that does not overlap another reflects a statistically significant difference. We also used the χ^2 ^test to estimate differences and we identify very small *P *values with '*P *<< 0.001' (which was generally the case), although the present study was not conducted to test any hypothesis. The validity of the questionnaire was tested in a group of local intensive care physicians (*n *= 18) and a group of medical students (*n *= 68) before and after a course on medical ethics given during the third term. Apart from sex and age, we also evaluated (as background variables) the participants' experiences of health care as a patient and as a relative, which were ranked as mainly positive, mainly negative, mixed positive and negative, or no experience.

## Results

Among physicians the response rate was approximately 70%, and among the general public it was about 51%. There were no differences in age and sex between responders and nonresponders. No difference was observed between the two groups in terms of their experiences of the health care system as a relative of a patient (Table 5), but there was a significant difference between groups in their experiences as a patient (*P *<< 0.001). Specifically, more members of the general public had experience as a patient, mixed positive and negative, whereas physicians had less experience of the health care system as a patient. Physicians tended to respond more promptly than members of the general public, but we found no differences in response pattern between those who responded immediately and those responding after one or two reminders. Almost 95% of neurosurgeons and most of the intensive care physicians were male; these majorities contrast with the 50:50 split among the general public. Analysis shows that the differences are due to group affiliation and not differences in sex distribution.

**Table 5:** Distribution of age and sex in doctors and members of the general public

	Doctors (*n *= 289)	Public (*n *= 501)	*P *value
Age (years; mean)	46.1	47.0	NS
Sex (male/female; *n*)	218/71	245/256	<<0.001
First responders (%)	64.1%	50.8%	
Second responders (1st reminder; %)	20.6%	24.6%	
Third responders (2nd reminder; %)	15.3%	24.6%	0.0007
Experience of health care as a patient (%)			0.000003
Positive	55.0%	50.9%	
Negative	1.4%	2.0%	
Both positive and negative	24.8%	38.8%	
No experience	18.8%	8.2%	
Experience of health care as a relative (%)			NS
Positive	42.6%	46.8%	
Negative	3.6%	3.8%	
Both positive and negative	45.2%	39.5%	
No experience	8.6%	9.9%	

Shown is the distribution of age and sex in doctors and members of the general public. The table also provides the rates of those who responded to the first and second reminders as well as the responders' experiences of health care either as a patient or as a relative.

When evaluating the specific arguments presented in the case, the internal dropout rate(respondents who have returned the questionnaire but answered partly) was low, but when estimating which of the arguments applied in a specific situation, the internal dropout rate was higher. In the first step, surgery or not, (Table 2), 84% (244/289) of physicians and 86% (433/501) of members of the general public answered; corresponding percentages for the second step, withdrawing ventilator treatment, were 89% (256/289) and 87% (435/501), and for the third step, giving sedatives and potent analgesics, they were 93% (268/289) and 87% (434/501). A few respondents identified two arguments as being the most important when they were asked to prioritize them; in such cases, both arguments were identified as being most important.

### Should neurosurgery be performed?

When prioritizing the arguments, most members of the public (59.8%) supported arguments in favour of performing the surgery, whereas a majority of the doctors (82.3%) were against surgery (Table 2); this difference was statistically significant (*P *<< 0.001). The most important value-based argument emphasized by the general public was that 'the primary task of health care is to save lives'. The empirically based arguments were the reference to 'the personal experience of the neurosurgeon' and 'the [lack of] quality of life'; the general public gave priority to the first argument, whereas the physicians stressed the latter. Neither group was swayed by the age of the patient and cost-benefit argument for not performing surgery, and there was little support for the son's wish that the surgery be performed or for regarding the withholding of surgery as a form of euthanasia.

### Should life-sustaining treatment be discontinued?

When evaluating the arguments for and against withdrawing life-sustaining treatment, most doctors (94.1%) and members of the general public (77.7%) deemed the most important arguments to be those against continuation of treatment. The prioritization of the arguments for withdrawing treatment differed significantly between groups, however (Table 3). The argument receiving the most support by both groups was that 'it only prolongs the death process', although significantly more physicians emphasized this argument (*P *<< 0.001). Although a minority in both groups believed that withdrawing ventilator treatment might be regarded as a form of euthanasia, significantly more of the general public attributed priority to this argument (*P *<< 0.001). A greater percentage of members of the general public also regarded adherence to the patient's wishes to be the most important argument (*P *<< 0.001).

### Should potent sedatives and analgesics be administered?

Most doctors (95.1%) and members of the general public (82%) agreed that potent sedatives and analgesics should be provided in the case presented. Significantly more physicians (*P *<< 0.001) were found to support this assertion when the arguments were specified (for example, that treatment should be provided even though it might hasten death; Table 4). It was also stressed by both groups that the intention should be to keep the patient calm and free from pain, and not primarily to hasten death, although some members of the general public felt that an intention to hasten death in the case presented is also acceptable. On the other hand, compared with doctors, significantly more members of the general public stated that potent sedatives and analgesics should not be provided if there were any risk for hastening death (*P *<< 0.001).

## Discussion

The most significant difference between the two groups is that concerning arguments in favour of performing neurosurgery in the case presented; the physicians were more reluctant to perform heroic surgery in the setting of a poor prognosis. However, as the case develops, differences in reasoning between doctors and members of the general public diminish somewhat, although they never quite disappear. On the whole, the results indicate that the general public has high expectations of what the health care system can achieve. Differences in judgement between the two groups result from divergent approaches to assessing empirical data and differences in moral reasoning.

### Validity factors

Among the doctors the response rate was not significantly lower than that in other similar studies. The fact that the response rate was rather low among members of the general public might be due to the nature of the six-page-long questionnaire focusing on special medical issues. Although the questionnaire was written using nontechnical terminology and was tested in pilot studies that included medical students in their third term, the issues at hand could have provoked strong emotions in potential questionnaire responders, such that only those who were interested in and capable of considering such questions actually responded. However, there was no difference between those who responded and nonresponders in terms of age and sex, and there was no differences in the response pattern between those who answered initially and those who responded to the second reminder. The two groups' experiences of health care as relatives of patients were rather similar, but the general public had greater experience of health care as a patient and had more combined positive and negative patient experiences. The age distributions of the two groups were similar, but there was a significant difference in sex distribution between groups; however, we only found statistical associations between the response patterns in the two groups, not between sex distribution within the groups. Differences in judgements might thus result from genuine differences between the two groups rather than sex bias.

The strengths of this study include the case-based questionnaire, which focuses on ethical reasoning and takes into account both fact and value judgements. By elucidating the ideas and expectations of the general public it might be possible to prevent miscommunication in future discussions with patients and relatives. Limitations of the study include the use of a vignette; these do not necessarily reflect real-life decision making. Accordingly, our findings should be interpreted in the light of other studies [12]. Furthermore, the rather low response rate among members of the general public mandates caution when interpreting the findings.

### Providing or withholding treatment

In agreement with the findings of other studies, physicians regarded quality of life considerations to be the most important argument in favour of withholding neurosurgery [7,11,13]. The ETHICATT study [7] also revealed that physicians and nurses were unlikely to emphasize the value of life *per se*, whereas patients were more inclined to prioritize this; these results are reflected in the present study. The important issue is whether public support for a proactive attitude is based on somewhat unrealistic expectations about the capability of medicine and the health care system, or whether other concerns should be taken into consideration. Evidence-based findings are now available that indicate that neurosurgical interventions in such cases are rather futile [14], but when the present study was conducted no such evidence existed. Only when treatment is clearly futile can its withholding be deemed ethically acceptable, and in such cases it is ethically equivalent to withdraw life-sustaining treatment [12]. If the outcome of such treatment is deemed to be uncertain (the personal experience of a neurosurgeon might influence this assessment), then it could be proposed that treatment be initiated and later withdrawn if it becomes clear that the intervention is futile. Withholding treatment that is not clearly futile is ethically more controversial than withdrawing clearly futile treatment. Such considerations might account for why significantly more of members of the general public regarded withholding life-sustaining treatment to be a kind of euthanasia, as compared with withdrawing such treatment. The significant difference between physicians and the public indicates that health care providers should be aware of how the general public reason in such cases.

Both physicians and members of the general public agreed that the patient's previously stated wishes is an important issue, but doctors were not inclined to consider the wishes of the patient's son, whereas most the public supported the value of the son's wishes.

Finally, it is interesting that neither members of the general public nor physicians considered the cost of such an operation or the age of the patient. These considerations may be taboo specific to Sweden, and responders may provide socially conventional answers. In the daily routine of an intensive care unit, both priority-settings (I. a. "Age alone is not relevant for decision of treatment" in Sweden) and age are relevant considerations [11] and are deemed standard factors to include in such studies [12].

### Withdrawing life-sustaining treatment

Discontinuing treatment because it only prolongs the dying process was regarded by both groups to be the most important argument. Treatment that prolongs the dying process might be interpreted as futile and as violating the patient's dignity. The most important issue, however, is whether discontinuing the treatment might be interpreted as equivalent to accepting a hastened dying process. Furthermore, is it reasonable to view discontinuing life-sustaining treatment and thus hastening death as a form of euthanasia? Even though most doctors would reject the association of treatment discontinuation with the concept of euthanasia, more than one-quarter of the public accept that such an association exists.

Although both groups appeared to be keen to respect the patient's previously stated wishes, more members of the general public deemed this to be the most important argument. One explanation for this difference might be that in Sweden advanced directives have no legal status, either in writing or orally. Furthermore, the wishes of a relative, at least in terms of demanding treatments, are considered but rarely acted upon. It is interesting to consider whether the existence of a written advanced directive would have changed the response pattern, and been considered by physicians and the son.

### Providing treatment that might hasten death

In the presentation of this case, it is stated that providing potent sedatives and analgesics could affect the respiratory centre and thus hasten death in a terminally ill patient. It is known that medical treatment can have two predictable effects: a desirable, positive one and an undesirable, harmful one. If we are unable to obtain the positive effect without also incurring the adverse one, then we face an ethical dilemma. However, if the overriding intention is to obtain the desirable effect (namely, to keep the patient symptom free), then it is considered acceptable to provide such a treatment, even though a predictable harmful and inevitable adverse effect might occur (hastening death); this reasoning is usually referred to as the 'principle of double effect' [15].

When they were presented with the case, all respondents were told about the potential harmful adverse effects of the drugs provided, and it is interesting that majorities of both groups apparently agreed with application of the principle of double effect in this specific case, and even attributed to it the highest priority. That we are actually dealing with a dilemma is reflected in the response pattern, with majorities of both groups supporting the argument that the drugs should not be provided if the purpose is to hasten death, even though both give little priority to the argument. Compared with the physicians, members of the general public were significantly more inclined to support the argument that drugs should be provided without risking a hastening of death. This could suggest that acceptance of the principle of double effect is more widespread among doctors than among members of the general public.

## Conclusion

The present study indicates that significant differences exist between physicians and the general public in how they reason in critical care situations. The discrepancies apparently result from different assessments of empirical facts and even differences in basic values. In order to avoid unnecessary dispute and miscommunication, doctors must better understand the nature of the views held by the general public (and hence those of patients' relative), and their expectations and preferences.

## Key Messages

Physicians are inclined to withhold treatment from a hopelessly ill patient, whereas most members of the general public tend to recommend it.

Majorities of both physicians and members of the general public are in favour of withdrawing life-sustaining treatment from a hopelessly ill patient.

Physicians and members of the general public forward different arguments for action and inaction when reasoning on the withholding and withdrawing of life-sustaining treatment.

In order to avoid miscommunication with patients and their relatives, physicians should be aware of their reasoning.

## Competing interests

The authors declare that they have no competing interests.

## Authors' contributions

Both authors contributed equally to the manuscript. AR made the first draft.
